# Speech Markers of Parkinson’s Disease: Phonological Features and Acoustic Measures

**DOI:** 10.3390/brainsci15111162

**Published:** 2025-10-29

**Authors:** Ratree Wayland, Rachel Meyer, Kevin Tang

**Affiliations:** 1 Department of Linguistics, University of Florida, Gainesville, FL 32611, USA; rmeyer2@ufl.edu (R.M.); kevin.tang@hhu.de; 2 Department of English Language and Linguistics, Institute of English and American Studies, Faculty of Arts and Humanities, Heinrich Heine University Düsseldorf, 40225 Düsseldorf, Germany

**Keywords:** Parkinson’s disease, speech biomarkers, deep neural network

## Abstract

**Background/Objectives:** Parkinson’s disease (PD) affects both articulatory and phonatory subsystems, leading to characteristic speech changes known as hypokinetic dysarthria. However, few studies have jointly analyzed these subsystems within the same participants using interpretable deep-learning-based measures. **Methods**: Speech data from the PC-GITA corpus, including 50 Colombian Spanish speakers with PD and 50 age- and sex-matched healthy controls were analyzed. We combined phonological feature posteriors—probabilistic indices of articulatory constriction derived from the Phonet deep neural network—with harmonics-to-noise ratio (HNR) as a laryngeal measure. Linear mixed-effects models tested how these measures related to disease severity (UPDRS, UPDRS-speech, and Hoehn and Yahr), age, and sex. **Results**: PD participants showed significantly higher [continuant] posteriors, especially for dental stops, reflecting increased spirantization and articulatory weakening. In contrast, [sonorant] posteriors did not differ from controls, indicating reduced oral constriction without a shift toward more open, approximant-like articulations. HNR was predicted by vowel height and sex but did not distinguish PD from controls, likely reflecting ON-medication recordings. **Conclusions**: These findings demonstrate that deep-learning-derived articulatory features can capture early, subphonemic weakening in PD speech—particularly for coronal consonants—while single-parameter laryngeal indices such as HNR are less sensitive under medicated conditions. By linking spectral energy patterns to interpretable phonological categories, this approach provides a transparent framework for detecting subtle articulatory deficits and developing feature-level biomarkers of PD progression.

## 1. Introduction

Parkinson’s disease (PD) is a complex neurodegenerative disorder characterized by intracellular *α*-synuclein aggregation and progressive loss of dopaminergic neurons in the substantia nigra pars compacta [1]. It is the second most common neurodegenerative disease after Alzheimer’s and the fastest-growing neurologic disorder, affecting over six million people worldwide [2]. While the principal clinical hallmarks are motor—resting tremor, rigidity, and bradykinesia—PD also entails a wide range of non-motor symptoms [1]. Among the most prevalent are speech and voice impairments, often manifesting as hypokinetic dysarthria. Close to 90% of individuals with PD experience voice disorders, while articulation and fluency problems occur in approximately 45% and 20% of cases, respectively [3]. These impairments are pervasive and progressive and may even precede the onset of motor symptoms [1].

Both laryngeal and supralaryngeal subsystems are affected in PD speech. At the laryngeal level, speakers frequently produce incomplete vocal fold closures, spirantized realizations (i.e., stops weakened toward fricatives), shortened stop closures, reduced intraoral pressure, and abnormal voice-onset times (VOTs), with effects varying across place of articulation and vowel contexts [4,5,6,7,8,9,10,11,12,13,14,15]. Evidence further suggested that coronal consonants (produced with the tongue tip or blade, e.g., [t, d]) are disproportionately vulnerable in PD and related dysarthrias [8,9,10,16]. Vowel articulation is likewise compromised, with reduced vowel space area, centralization of formants, and reduced consonant-vowel coarticulation [17,18,19,20,21]. Traditional acoustic studies have relied on measures such as closure duration, VOT, and F1/F2 formants [13,14,15,22], while more recent approaches apply computational methods ranging from recurrent networks trained to output phonological feature posteriors (probabilities for features such as [continuant], distinguishing stops from fricatives, or [sonorant], distinguishing obstruents from vowels/nasals/liquids) [23] to broader learned embeddings [24,25], providing interpretable and data-driven indices of articulatory categories.

At the laryngeal level, PD speech exhibits changes in both voice quality and prosody. In complete glottal closure, asymmetrical vibration, and reduced respiratory drive yield elevated jitter and shimmer, reduced cepstral peak prominence, and lower HNR [26,27,28,29,30,31]. Sustained vowels are often analyzed for this purpose, though continuous speech may reveal similar information [32]. Prosodic abnormalities, including reduced pitch variability, monoloudness, and altered timing patterns, are also widely reported [33]. Other studies have explored prosody in relation to medication effects, differential diagnosis, and domain specificity [34,35,36]. Taken together, these findings highlight PD speech as multidimensional, with articulatory and phonatory deficits contributing in complementary but distinct ways. Yet relatively few studies have jointly examined these subsystems in the same cohort or directly modeled their relationship to clinical severity [6,7,8].

The present study addresses this gap by analyzing Colombian Spanish speech from individuals with PD and matched controls, combining articulatory correlates (Phonet-based posteriors) and acoustic correlate of phonation (HNR from sustained vowels). This dual approach allows us to test whether different clinical scales are differentially sensitive to articulatory versus phonatory decline, and to evaluate the potential of feature-based representations for capturing PD speech impairments across subsystems.

## 2. Approaches to PD Speech Analyses

Research on PD speech has advanced along three complementary trajectories. Early studies focused on handcrafted acoustic features, grounded in speech science and directly interpretable in terms of articulatory or phonatory mechanisms. More recent work has shifted toward machine learning-based analyses, which automatically extract high-dimensional patterns from the signal, offering scalability and strong classification performance but often at the cost of interpretability. Bridging these two approaches, phonological feature posteriors provide an intermediate framework that combines the representational power of learned models with the transparency of linguistically motivated categories such as [voice], [continuant], or [sonorant]. The subsections below review these major analytical approaches to PD speech and situate the present study within this framework.

### 2.1. Overview of Analytical Approaches

Early analyses of PD speech relied on handcrafted acoustic measures that map cleanly onto articulatory and phonatory mechanisms. Temporal indices such as VOT and closure duration, spectral cues such as vowel formants and vowel-space area, and voice-quality metrics such as jitter, shimmer, HNR, and cepstral-peak prominence remain foundational [17,28,37]. These interpretable features correlate with clinical severity—sustained-vowel dysphonia measures significantly predict UPDRS scores [28], and vowel-space reduction tracks disease progression and treatment response [16]. However, handcrafted approaches require manual segmentation and typically evaluate cues in isolation, even though PD speech reflects multidimensional reorganization. For instance, stop lenition involves concurrent changes in intraoral pressure, closure duration, spectral tilt, and VOT, while reduced prosodic variability arises from coupled shifts in F_0_ range, intensity, and timing. Capturing such interdependent adjustments motivated the adoption of more integrative computational frameworks.

Machine learning approaches extended these analyses by offering scalability and automation. Early studies combined engineered features (e.g., MFCCs, formant- and prosody-based statistics) with classifiers such as SVMs or random forests, achieving high within-language accuracy for PD detection [38,39,40]. Large-scale reviews [41,42] confirmed their diagnostic promise but highlighted barriers to generalization and limited physiological interpretability. Subsequent deep-learning (DL) and self-supervised learning (SSL) frameworks learned directly from spectrograms or raw waveforms [24,41]. Pretrained models such as WavLM and Wav2Vec 2.0 improved robustness across corpora [43,44], and newer architectures aligned SSL embeddings with interpretable articulatory or phonatory streams [45,46]. While these models achieve strong accuracy, their “black-box” nature limits clinical uptake, emphasizing the need for explainable representations that preserve linguistic grounding.

Bridging interpretability and performance, phonological feature posteriors extend acoustic analysis by reinterpreting the acoustic properties of the speech signal in articulatory–phonological terms that reflect how features like [continuant] (degree of oral constriction), [sonorant] (degree of vocal tract resonance), and [voice] (presence of vocal-fold vibration) shape speech production. Neural networks trained to estimate the probabilities of these features capture both categorical contrasts and gradient realizations [23]. This representation aligns naturally with processes like Spanish lenition—a shift from [−continuant] to [+continuant] or [−sonorant] to [+sonorant]—which parallels the articulatory weakening observed in PD speech [4,47]. By grounding learned models in articulatory principles, posterior-based approaches provide a transparent bridge between acoustic analysis and clinical interpretation, motivating the use of Phonet—the feature-posterior framework adopted in the present study.

### 2.2. Phonet

Phonet, first proposed by [23], operationalizes phonological posteriors by using neural networks to estimate the probability that each short time frame expresses articulatory properties such as [continuant], [sonorant], or [voice]. The model is phonologically motivated and language-specific, requiring a well-defined phonological feature set and a segmentally aligned acoustic corpus (typically obtained via forced alignment). In contrast to generic deep embeddings that yield opaque high-dimensional vectors, Phonet maps acoustic information onto features with clear phonological interpretation, linking acoustic variation directly to articulatory categories.

Technically, Phonet employs bidirectional recurrent neural networks with gated recurrent units (GRUs) trained on log Mel-filterbank energies from short spectral windows. This architecture models context from both past and future frames, capturing coarticulatory effects and temporal dependencies without requiring explicit duration measures. The output is a sequence of posterior probabilities that reflect both categorical differences (e.g., stop vs. fricative) and gradient realizations (e.g., degrees of lenition).

While ref. [23] introduced Phonet as a general framework for pathological speech analysis, early applications of phonological posteriors to Parkinsonian speech predate Phonet. For example, ref. [48] used differential phonological posterior features to characterize voice quality in PD, showing that these features could quantify breathy, creaky, tense, falsetto, and harsh phonation components and predict dysarthria severity as rated by the Frenchay Dysarthria Assessment. More recently, ref. [49] applied the Phonet toolkit directly to classify Spanish-speaking PD patients versus healthy controls, achieving high classification accuracy and confirming the feasibility of using feature-level posteriors for clinical assessment.

Phonet has since been validated and applied across multiple domains. It has been used to quantify lenition in Spanish voiced stops [50,51,52], to model PD-related speech impairments in Spanish [53], to characterize segmental drift in intoxicated speech [54] and to track developmental phonetic changes in L2 Spanish learners [55]. Across these applications, Phonet’s posterior outputs have proven to be a robust and interpretable index of gradient articulatory variation, bridging neural modeling with linguistic theory and clinical practice.

## 3. This Current Study

Most prior research on PD speech has focused either on segmental articulation or on global voice quality, but rarely on both within the same dataset, and rarely using feature-based methods that directly model phonological properties. Consequently, little is known about how deficits in the supralaryngeal system (articulatory control of consonants) and the laryngeal system (phonatory control of sustained vowels) unfold in tandem, or how these deficits relate to clinical severity scales and demographic factors.

The present study addresses these gaps using speech from Colombian Spanish speakers with PD and matched healthy controls. We compare supralaryngeal measures—continuant and sonorant posterior probabilities for stops—with a laryngeal measure (HNR) for sustained vowels—across tasks and participant groups. By linking these measures to UPDRS, UPDRS-speech, and Hoehn and Yahr scores, we test which clinical scales best predict acoustic variation and whether articulatory and phonatory domains differ in their sensitivity to disease progression. This integrated approach provides a comprehensive account of PD-related deficits across representational levels, from segment-specific articulatory properties to laryngeal-phonatory control.

### 3.1. Research Questions and Hypotheses

Building on the evidence reviewed above, the study examines how supralaryngeal and laryngeal speech measures relate to PD status and clinical severity. Our goals are twofold: (i) to determine whether articulatory and phonatory deficits exhibit distinct patterns in individuals with PD, and (ii) to assess which commonly used clinical rating scales are most sensitive to these deficits.

**RQ1.**  *How do supralaryngeal measures—continuant and sonorant posterior probabilities for stop consonants—and laryngeal measures—harmonics-to-noise ratio (HNR) for sustained vowels—differ between PD participants and healthy controls, and are these group differences modulated by place of articulation or vowel height? By focusing on speech-based articulatory measures, this study complements prior work using nonspeech oral-motor tasks, which have shown limited ability to predict consonant imprecision in PD [56].*

**H1.**  *PD participants are predicted to produce stop consonants with higher continuant posterior probabilities, reflecting greater lenition (i.e., spirantization of stops into fricatives). This prediction is supported by kinematic evidence showing reduced amplitude and velocity of lower-lip and jaw movements in PD during sentence-level speech [7] and scaled-down tongue movements and slower vowel-related gestures in connected speech [6]. Phonological feature posteriors offer a promising, interpretable way to quantify this articulatory hypokinesia in a manner that is both sensitive and interpretable.*

**H2.**  *PD participants are expected to produce sustained vowels with lower HNR values than healthy controls, reflecting incomplete glottal closure and increased turbulent noise, as documented in acoustic studies of dysphonia and PD speech [26,27]. Furthermore, analyses of sustained vowels in both healthy and dysphonic speakers show that HNR tends to be lower for low vowels such as /a/ and higher for high vowels such as /i/and /u/ [57,58], suggesting that vowel height modulates periodic energy even in non-disordered speech. We therefore predict systematic vowel-height effects on HNR, with larger PD-control differences for high vowels than for low vowels.*

**RQ2.**  *Which clinical severity metric—Unified Parkinson’s Disease Rating Scale (UPDRS), UPDRS-speech, or Hoehn and Yahr (H–Y) stage—most strongly predict variation in supralaryngeal and laryngeal measures, and are these relationships influenced by participant age or sex?*

**H3.**  *UPDRS-speech scores are expected to be the strongest predictors of supralaryngeal measures, as this subscore specifically targets speech-motor function. The UPDRS speech item correlates moderately with perceptual speech impairment across voice, articulation, prosody, and fluency domains, indicating that it captures articulatory changes more sensitively than the overall UPDRS total score [59].*

**H4.**  *H–Y is expected to predict laryngeal measures (e.g., HNR) more strongly than supralaryngeal ones, reflecting the effect of overall motor severity on glottal closure and phonatory stability. H–Y stage has been shown to be significantly associated with global speech impairment [59] and to substantially improve the variance explained in models of speech intelligibility when included as a covariate (≈54% increase) [60]. Although a direct relationship between H–Y and HNR has not been systematically reported, the documented influence of disease stage on phonatory measures makes such an association plausible.*

### 3.2. Materials and Methods

#### 3.2.1. Data

Speech data were drawn from the PC-GITA corpus, a publicly available dataset of Colombian Spanish speakers performing a range of speech-based tasks [61]. Participants included 50 individuals with Parkinson’s disease (PD; 25 men, 25 women) and 50 age- and sex matched healthy controls, all native speakers of Colombian Spanish. Ages ranged from 33–77 years for men (PD M = 62.2 ± 11.2; controls M = 61.2 ± 11.3) and 44–75 years for women (PD M = 60.1 ± 7.8; controls M = 60.7 ± 7.7) [61]. All PD participants were evaluated by an expert neurologist and recorded in the ON-medication state (≤3 h after their morning dose). Clinical ratings available in the corpus include the Unified Parkinson’s Disease Rating Scale (UPDRS)—a multidimensional clinical scale of motor and non-motor symptoms, with the speech subscore specifically evaluating voice, articulation, and prosody—and the Hoehn and Yahr (H–Y) staging scale, which classifies disease severity from stage 1 (unilateral symptoms) to stage 5 (severe disability). These measures were used as predictors in our analyses. At the time of recording, patients also received ratings according to the MDS-UPDRS III [62], with an average score of 36.6, confirming moderate motor involvement and ensuring clinical consistency of the dataset.

We analyzed data from three tasks: a sentence reading task targeting all six Spanish stops (/p, b, t, d, k, ɡ/); the “pataka” diadochokinetic (DDK) task targeting the three voiceless stops; and a sustained vowel task in which participants produced each vowel (/a, e, i, o, u/) three times for as long as possible. Table 1 provides counts of stop tokens by group.

#### 3.2.2. Measures

To assess how articulatory and phonatory deficits relate to disease progression, we examined three standard clinical measures of PD severity: the UPDRS) [62], the UPDRS-speech subscore, and the H–Y scale [63]. The UPDRS is a continuous measure ranging from 0 to 200, based on 50 items that evaluate motor and cognitive abilities. Each item is rated from 0 (normal) to 4 (severe), with higher totals reflecting greater impairment. The UPDRS-speech is a single item within the UPDRS that specifically assesses volume, modulation of prosody, and clarity, including slurring, palilalia, and tachyphemia, and is scored on the same 0–4 scale. The Hoehn and Yahr scale is a widely used index of overall disease progression scored from 0 to 5, with intermediate stages 1.5 and 2.5; values from 1 to 3 indicate mild to moderate disability, while values from 4 to 5 indicate moderate to severe disability and loss of independence.

Table 2 summarizes the distribution of scores in PC-GITA. On the UPDRS, the mean score was 37.7 (SD = 18.3, range = 6–93). The UPDRS-speech scores averaged 1.34 (SD = 0.82, range = 0–2), indicating at most mild speech impairment. The H–Y scores averaged 2.19 (SD = 0.66, range = 0–4), which corresponds to bilateral involvement with early balance difficulties but overall mild to moderate disability. These values indicate that participants were largely in the early to middle stages of disease progression, with few scoring in the severe range on any of the rating scales.

#### 3.2.3. Analysis

The data were aligned using the MFA Spanish forced aligner (v.2.0.0) [64] and then inferenced on the trained Phonet model in 10 ms frames [50]. The posterior probability of the [continuant] and [sonorant] features of the six stops was extracted for data analysis. These features were chosen because they capture weakening of the oral constriction gesture, the articulatory dimension most affected in PD. The feature [voice] was not included because voicing contrasts in Spanish stops are largely context-dependent and therefore not a reliable index of articulatory weakening in this dataset. Likewise, [nasal] was not analyzed because the study focused on oral stops rather than nasal consonants. Thus, [continuant] and [sonorant] provide a minimal, theory-consistent feature set optimized for detecting gradient weakening of tongue gestures in stop production. HNR data were gathered from Praat (v. 6.4.14) using a script [65]. Data was analyzed with linear mixed effects models in R (4.4.1) [66]. Three separate models were fit with each of the neural network outputs (continuant posterior probability, sonorant posterior probability, and HNR) as the dependent variable. Fixed predictors included the three Parkinson’s disease rating scales (UPDRS, UPDRS-speech, and Hoehn and Yahr), participant sex, and age. For both posterior models, place of articulation (bilabial, dental, velar) was also included for the HNR model, vowel height (open, mid, close) was included. Group (PD vs. control) was not entered as a main effect because the clinical rating scales only apply to PD participants and therefore collinear with group. However, interactions involving group and participant factors (group × place, group × sex, group × age, and sex × age) were tested. Speaker was included as a random slope to account for within-subject variability. Categorical predictors were contrast-coded to ensure interpretable parameter estimates. Factors with two levels (group, sex) were deviation-coded (values −0.5 and +0.5) such that the intercept represented the grand mean and coefficients reflected deviations from it. Factors with more than two levels (place, UPDRS speech, H–Y) were forward difference-coded, comparing each level to the mean of subsequent levels. Continuous predictors (age and UPDRS scores) were entered as continuous numerical variables.

## 4. Results

### 4.1. Continuant Posterior Probability

**Figure 1:**
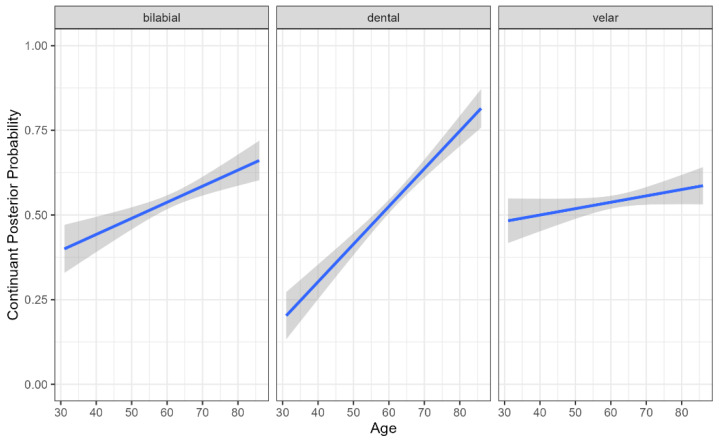
Continuant Posterior Probability by Participant Age and Stop Place of Articulation. Higher continuant probabilities indicate greater spirantization, reflecting more fricative-like realizations of stop consonants.

**Figure 2:**
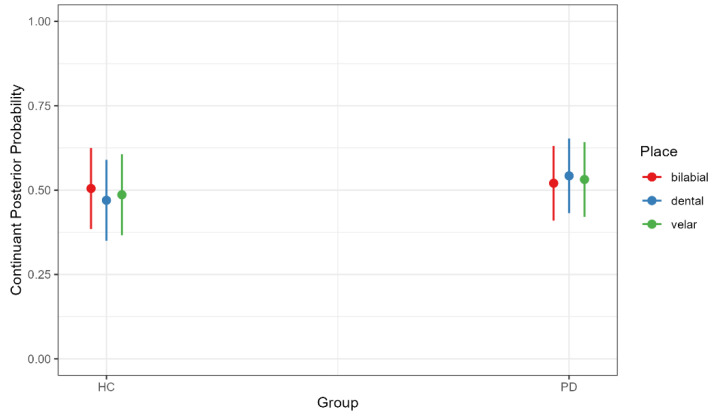
Continuant Posterior Probability by Participant Group (healthy controls [HC] and Parkinson’s diseases [PD]) and Stop Place of Articulation. Higher continuant probabilities indicate greater spirantization, reflecting more fricative-like realizations of stops across places of articulation.

There was a significant main effect of age, with older participants exhibiting a higher continuant posterior probability regardless of PD diagnosis (b = 0.660, SE = 0.002, t = 3.877, *p* < 0.001), indicating a greater degree of lenition (spirantization). There was also an effect of place, with bilabial (b = 0.381, SE = 0.090, t = 4.236, *p* < 0.001) and velar stops (b = −0.550, SE = 0.090, t = −6.081, *p* < 0.001) produced with higher continuant posterior probabilities than dental stops (Figure 1). However, this effect was modulated by an interaction between group and place. Although neither group comparison reached significance, the pattern of means differed: in the HC group, bilabial stops had slightly higher continuant posterior probabilities than dental stops (b = 0.035, SE = 0.019, t = 1.869, *p* = 0.422), whereas in the PD group, the pattern was reversed, with dental stops showing numerically higher values than bilabials (b = −0.022, SE = 0.018, t = −1.208, *p* = 0.833) (Figure 2). This nonsignificant trend may point to a greater susceptibility of tongue-tip articulations in PD, a possibility that merits investigation in larger samples.

**Figure 3:**
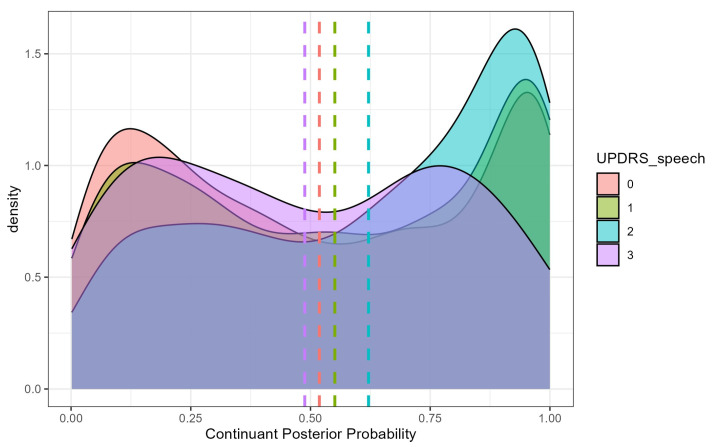
Continuant Posterior Probability by UPDRS-speech rating among Parkinson’s disease (PD) participants. Higher continuant probabilities indicate greater spirantization, reflecting more fricative-like realizations of stop consonants with increasing speech-severity scores.

Among the clinical severity metrics, only the UPDRS-speech score significantly predicted continuant posterior probability. PD participants with a score of 1 produced stops with lower continuant posterior probabilities than those with a score of 2 (b = −0.141, SE = 5.189, t = −2.199, *p* = 0.031), indicating that more severe speech ratings were associated with a stronger degree of lenition (Figure 3). Finally, there was a significant place × age interaction. The slope of age-related change was steepest for dental stops, with continuant posterior probability increasing more rapidly with age than for bilabials (b = −0.006, SE = 0.001, t = −4.211 *p* < 0.001) or velar (b = 0.008, SE = 0.001, t = 6.109, *p* < 0.001), suggesting that age-related lenition effects are most pronounced for tongue-tip articulations.

### 4.2. Sonorant Posterior Probability

**Figure 4:**
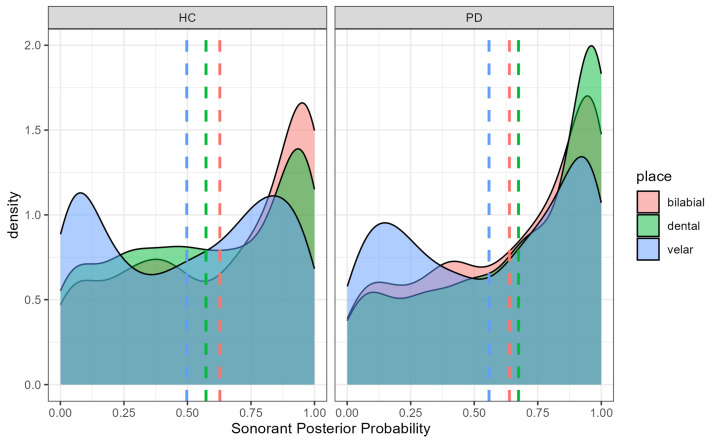
Sonorant Posterior Probability by Participant Group and Stop Place of Articulation. Higher sonorant probabilities reflect more vowel- or approximant-like realizations of stop consonants.

The effect of place of articulation on sonorant posterior probability paralleled that observed for continuant probability. Dental stops had lower sonorant posterior probabilities than bilabials (b = 0.0203, SE = 0.086, t = 2.369, *p* = 0.018) or velar (b = −0.169, SE = 0.085, t = −1.976, *p* = 0.048) stops (Figure 4). However, the place × group interaction revealed a slightly different pattern than for continuant posteriors. In the healthy control group, bilabial stops had significantly higher sonorant posterior probabilities than dental stops (b = 0.054, SE = 0.018, t = 3.034, *p* = 0.029). In the PD group, however, this place-based difference was not significant (b = −0.031, SE = 0.017, t = −1.801, *p* = 0.465). Although the place effect for sonorants was weaker than for continuants, the pattern still suggests relatively lower sonorant probabilities for dentals across groups, consistent with greater articulatory constriction for tongue-tip gestures. When interpreted alongside the continuant results, these findings suggest that PD speakers tend to lenite (spirantize) dental stops toward fricatives (higher [continuant]) but not toward sonorants (no systematic increase in [sonorant] probability).

**Figure 5:**
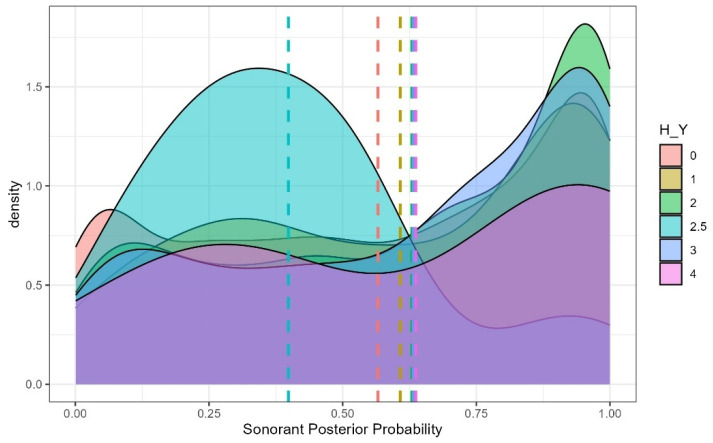
Sonorant Posterior Probability by Hoehn and Yahr Score. Higher sonorant probabilities reflect more vowel- or approximant-like realizations of stop consonants.

**Figure 6:**
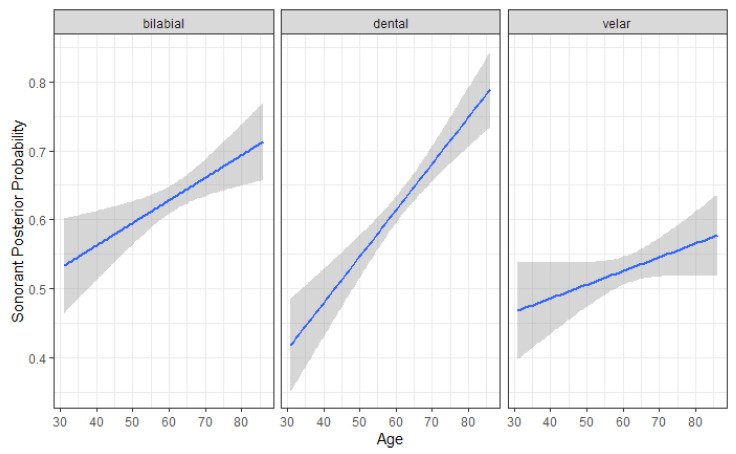
Sonorant Posterior Probability by Participant Place of articulation (bilabial, dental, velar) and Age. Higher sonorant probabilities reflect more vowel- or approximant-like realizations of stop consonants.

Among clinical severity metrics, Hoehn and Yahr (H–Y) stage predicted sonorant posterior probability, with a significant increase observed between levels 2.5 and 3 (b = −0.040, SE = 0.220, t = −2.004, *p* = 0.048). A significant place × sex interaction was also observed: among women, dental stops had higher sonorant posterior probabilities than velar stops (b = 0.150, SE = 0.017, t = 8.790, *p* < 0.001), whereas no reliable place differences were observed in men (b = 0.030, SE = 0.018, t = 1.652, *p* = 0.564) (Figure 5). Finally, a significant place × age interaction was found, with dental sonorant values increasing more steeply with age than bilabial (b = –0.003, SE = 0.001, t = −2.259, *p* = 0.024) or velar (b = 0.004, SE = 0.001, t = 3.058, *p* = 0.002) stops (Figure 6).

### 4.3. Harmonics-to-Noise Ratio 

**Figure 7:**
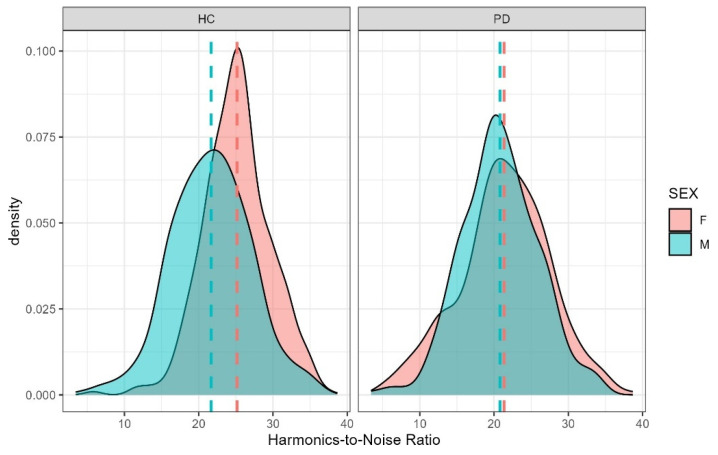
Harmonics-to-Noise Ratio (HNR) by Participant Sex and Group. Higher HNR values reflect relatively more periodic, harmonically rich voice quality and more regular vocal-fold vibration.

**Figure 8:**
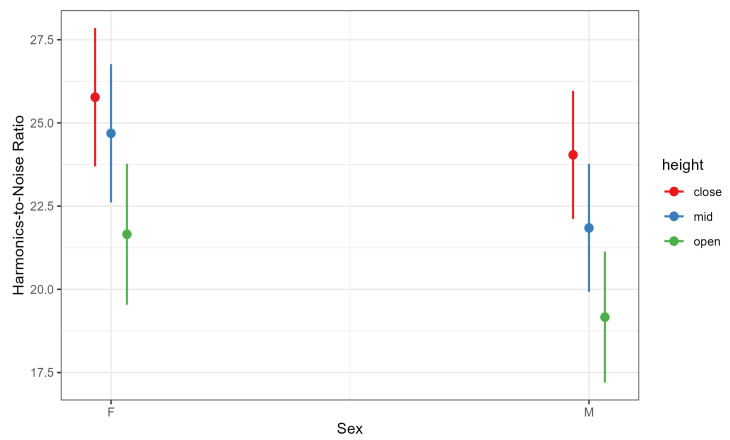
Harmonics-to-Noise Ratio (HNR) by Participant Sex and Vowel Height. Higher HNR values reflect relatively more periodic, harmonically rich voice quality and more regular vocal-fold vibration.

Harmonics-to-noise ratio was significantly predicted by both vowel height and participant sex. As expected, mid vowels had higher HNR values than low vowels (b = 3.960, SE = 1.710, t = 2.316, *p* = 0.021), consistent with prior evidence that HNR increases with vowel height [67]. Unexpectedly, women in our sample produced higher HNR values than men (b = −12.516, SE = 5.481, t = −2.284, *p* = 0.025), despite previous reports that men typically exhibit more harmonically rich voices [68] (Figure 7). The vowel-height × sex interaction indicated that the sex difference was not significant for close vowels (b = 1.732, SE = 0.770, t = 2.251, *p* = 0.224) but emerged for mid (b = 2.843, SE = 0.770, t = 3.694, *p* = 0.005) and open vowels (b = 2.490, SE = 0.825, t = 3.020, *p* = 0.035), with women showing higher HNR values than men for these vowel categories (Figure 8). These findings partially support H2, confirming the expected vowel-height effect on HNR while revealing sex-specific differences that may warrant further investigation.

## 5. Discussion

This study investigated how supralaryngeal and laryngeal speech measures differentiate individuals with Parkinson’s disease (PD) from healthy controls and how these measures relate to clinical severity ratings. By combining phonological feature posteriors for stop consonants with harmonics-to-noise ratio (HNR) for sustained vowels, we jointly analyzed articulatory and phonatory subsystems within a unified, interpretable framework. Unlike categorical phone-based analyses or black-box embeddings, the feature-posterior approach captures gradience in articulatory and phonatory control using phonologically motivated dimensions such as [continuant] and [sonorant], which reflect how airflow and constriction are coordinated during speech production.

Our findings support and extend previous evidence of articulatory weakening in PD. Specifically, PD participants showed significantly elevated [continuant] posteriors for stop consonants—particularly for dentals—consistent with increased spirantization, or fricative-like realizations of oral stops. In contrast, [sonorant] posteriors did not differ significantly from the healthy control group, indicating that the articulation changes reflects increased oral airflow through a still narrow constriction—consistent with turbulent, fricative-like airflow—rather than a transition toward more open, vowel-like articulations characteristic of approximants. This selective increase in [continuant]—without a corresponding rise in [sonorant]—suggests partial articulatory weakening consistent with early-to-mid-stage PD, where oral closures are reduced but not fully lost. These effects parallel aerodynamic findings of reduced intraoral pressure and incomplete stop closures in PD (e.g., [4]) and complement kinematic evidence of reduced amplitude and speed of tongue and jaw movements during both consonant and vowel production in PD [6].

Taken together, these results point to articulatory hypokinesia—a reduction in movement magnitude and precision—leading to weaker constriction gestures and greater oral airflow. Our feature-based analysis extends this literature by localizing the effect to tongue-tip (coronal) gestures, which showed the steepest increase in [continuant] probability. This place-specific vulnerability suggests that coronal articulations may serve as early indicators of neuromotor decline.

Age-related effects followed a similar pattern, with dental stops exhibiting the strongest increases in lenition with age. These findings align with prior proposals that articulatory involvement in PD progresses from posterior to anterior oral regions [14]. The fact that increased [continuant] posteriors appear in an on-medication, early-to-mid stage cohort suggest that coronal instability may persist despite dopaminergic treatment—or that feature posteriors are sensitive enough to detect subclinical weakening before overt articulatory errors emerge. In either case, our findings complement and extend [14]’s progression hypothesis by identifying a potential early marker of anterior articulatory involvement.

Because posterior probabilities are continuous, they provide a quantitative, high-resolution window into this biomechanical drift that may precede categorical misarticulations. Importantly, this articulatory pattern is not language-specific: coronal consonants are present in virtually all spoken languages and universally involve fine spatial and timing control of the tongue tip. Thus, the heightened sensitivity of coronal articulations observed here likely reflects a general biomechanical vulnerability of anterior tongue gestures, rather than a Spanish-specific pattern.

As expected, vowel height was a strong determinant of HNR, with mid vowels showing higher values than low vowels, and women exhibited higher HNR for mid and open vowels. However, contrary to some previous reports, HNR did not differentiate PD from controls or predict clinical severity in this early-to-mid-stage, ON-medication cohort. This partial support for our second hypothesis suggests that vowel-intrinsic and demographic factors dominate HNR variance under these conditions. Crucially, this null result should not be taken to imply an absence of laryngeal involvement. Large-scale perceptual studies have consistently found laryngeal impairment to be the most frequent and severe subsystem deficit in PD, with [14] reporting glottal dysfunction in nearly 90% of their cohort and often in isolation. A substantial body of acoustic work also demonstrates that phonatory impairment can emerge as one of the earliest detectable signs of PD. Using a database of sustained vowel phonations, [28] demonstrated that nonlinear dysphonia measures predict UPDRS motor and total scores with clinically meaningful accuracy, highlighting the sensitivity of glottal control as an early biomarker. Taken together, these findings indicate that the lack of HNR differences in our study likely reflects methodological factors—particularly the ON-medication recording state, which is known to partially normalize phonatory irregularities and improve some aspects of phonation and voice quality—and the limited diagnostic sensitivity of HNR as a single-parameter measure. HNR is influenced by vowel type, F_0_ range, and analysis bandwidth and may therefore fail to capture subtle irregularities when analyzed in isolation. Nevertheless, given that laryngeal deficits are among the most frequently reported impairments in PD, the absence of HNR differences here likely reflects individual and methodological factors—particularly disease-stage variability and the ON-medication recording state—rather than the absence of phonatory involvement. Longitudinal studies including OFF-medication recordings and multidimensional phonatory indices would help clarify this relationship.

Medication-related variability further contextualizes these findings. The absence of group differences in HNR should be interpreted in light of participants’ ON-medication status. Recent studies examining the effects of levodopa on PD speech have yielded mixed results: some report no significant OFF→ON changes in HNR or other hypokinetic-dysarthria dimensions in early PD, even when limb motor scores improve [69], and no benefit in late-stage PD despite motor improvement [70]. Others demonstrate partial normalization of prosodic, respiratory, and spectral speech features following levodopa intake and high classification accuracy for medication state using digital speech biomarkers [71]. Importantly, ref. [72] found that HNR improvements were strongest for /m/ and that combining /a/, /o/, and /m/ yielded the best discrimination of medication state. Taken together, these results suggest that the lack of HNR differences in our ON-medication cohort may reflect both dopaminergic attenuation of phonatory irregularities and the limited sensitivity of single-vowel HNR to medication effects, which may emerge more robustly in nasals, multiple phoneme contexts, or multidimensional acoustic measures. Future work should therefore evaluate multidimensional phonatory indices (e.g., cepstral peak prominence, spectral tilt, and glottal flow modeling), include OFF-medication recordings or longitudinal follow-ups, and expand phonatory tasks to multiple phonemes to better capture early-stage laryngeal dysfunction.

The contrast between our supralaryngeal and laryngeal findings also has implications for models of disease progression. Classic work by [14] proposed that oral articulatory involvement follows a posterior-to-anterior sequence, with tongue-tip dysfunction least common and likely to emerge later in the disease course. Our results refine this framework rather than contradict it. While numerous studies show that laryngeal impairment can appear very early—even at prodromal stages—our findings suggest that subtle coronal instability may arise in parallel with or shortly after these early phonatory changes, despite dopaminergic treatment. In other words, feature-posterior measures may capture a subclinical, pre-categorical stage of anterior articulatory weakening that precedes overt errors and would be missed by perceptual assessment. If confirmed in longitudinal data, this would extend the posterior-to-anterior progression model by adding an intermediate, subphonemic phase of anterior decline, indicating that coronal consonants may serve as sensitive early probes of supralaryngeal decline, complementing early laryngeal indicators.

The clinical analyses further clarify how these measures map onto disease severity. Among the clinical predictors, the UPDRS-speech subscore emerged as the strongest predictor of [continuant] posteriors, indicating that a speech-specific clinical rating aligns most closely with articulatory performance, consistent with prior evidence linking UPDRS-speech to acoustic and kinematic indices of articulatory control [73,74]. Hoehn and Yahr (H–Y) stage showed a more localized effect, predicting increased [sonorant] posteriors at the transition from stage 2.5 to 3. Because this stage marks the onset of bilateral motor involvement and early postural change, this association may indicate that advancing motor severity begins to affect oral constriction and voicing coordination. In contrast, neither H–Y nor the global UPDRS predicted HNR in our data, underscoring the limited sensitivity of global motor scales for detecting early, subtle phonatory change and highlighting the need for speech-specific or multidimensional laryngeal metrics.

Finally, our choice of Phonet and phonological feature posteriors as the analytic framework deserves emphasis. Unlike handcrafted acoustic cues that require segment-specific definitions or opaque learned embeddings that sacrifice interpretability, feature posteriors provide probabilistic, phonologically grounded indices by reinterpreting spectro-temporal energy (log-Mel-filter bank inputs) in articulatory terms. This approach allowed us to disambiguate spirantization from approximantization, quantify place-specific vulnerabilities, and trace age-related trajectories, all within a single unified representation. Methodologically, feature posteriors offer a bridge between the richness of machine learning representations and the interpretability required for clinical and linguistic applications. In doing so, they support the development of compact, clinically meaningful speech biomarkers—such as age-normalized dental [continuant] slopes—that can be deployed for telehealth monitoring or early screening.

Several limitations should be acknowledged. The PD cohort was skewed toward early-to-mid stages with limited UPDRS-speech variability (0–2), reducing the range of detectable associations. All recordings were ON medication, likely attenuating both articulatory and phonatory deficits. The stop dataset was unbalanced, with few /ɡ/ tokens and no voiced stops in the diadochokinetic task, constraining place contrasts. Additionally, because the data were drawn exclusively from Colombian Spanish speakers, the findings may not fully generalize to other languages or dialects with different stop inventories, phonotactic patterns, or prosodic characteristics. Extending this approach to other corpora (e.g., PPMI, [75]) or using multilingual feature-extraction models could help evaluate the cross-linguistic robustness of feature-posterior analyses. Finally, HNR as a single laryngeal measure is coarse; richer multidimensional indices such as cepstral peak prominence, spectral tilt, and glottal flow parameters should be incorporated in future studies. Addressing these limitations in a longitudinal design will allow us to determine whether supralaryngeal deficits consistently precede perceptible laryngeal ones, or whether the two systems exhibit overlapping but dissociable timelines of decline.

## 6. Conclusions and Future Directions

To our knowledge, this is one of the first studies to combine feature-based articulatory and laryngeal measures in a single analysis of PD speech using the PC-GITA corpus. Our results show that articulatory indices derived from phonological features—particularly [continuant] posteriors for dental (tongue-tip) stops—are sensitive to both aging and PD-related decline, correlating most strongly with the UPDRS-speech subscore. In contrast, sustained-vowel HNR primarily reflected vowel-intrinsic and demographic variation, with limited sensitivity to clinical severity in this ON-medication, early-to-mid stage cohort. Together, these findings suggest that articulatory features may provide earlier or more reliable markers of disease impact than single-parameter phonatory measures. Importantly, they complement prior perceptual observations suggesting that subsystem involvement may progress from laryngeal to oral articulators, highlighting the value of featural analyses in detecting incipient articulatory instability even before categorical errors emerge.

Future work should expand this framework by incorporating a wider severity range, OFF- and ON-medication recordings with sufficient washout, and longitudinal follow-ups to track within-speaker trajectories over time. Task design should include balanced materials across voiced and voiceless stops, connected speech, and multiple sustained-phonation targets (e.g., /a/, /o/, and /m/) to maximize sensitivity to articulatory and phonatory changes [72]. Laryngeal assessment should move beyond single-parameter HNR to multidimensional indices such as cepstral peak prominence, spectral tilt, LTAS moments, and glottal flow modeling, complemented by prosodic and respiratory measures that may be more responsive to dopaminergic modulation [71]. Finally, integrating supralaryngeal features with these multidimensional laryngeal and prosodic measures could yield a composite speech-severity index aligned with UPDRS-speech, supporting remote monitoring, early diagnosis, and evaluation of treatment effects. By situating feature-based analyses at the interface of phonological representation and clinical assessment, this work lays the foundation for more sensitive, interpretable, and clinically faithful speech biomarkers of PD progression.

## Figures and Tables

**Table 1:** Counts of stop tokens produced by participants in the PC-GITA corpus (50 PD participants, 50 age- and sex-matched healthy controls).

Phone	Healthy Controls	Parkinson’s Patients
p	307	293
b	207	255
t	382	395
d	162	184
k	492	526
g	22	21
Total	1572	1674

**Table 2:** Clinical ratings for PD participants in the PC-GITA corpus. Means, standard deviations, and ranges are shown for the Unified Parkinson’s Disease Rating Scale (UPDRS), the UPDRS-speech subscore, and the Hoehn and Yahr (H–Y) scale.

Scale	Average Score	Standard Deviation	Range	Scale Range
UPDRS	37.660	18.315	6–93	0–200
UPDRS-speech	1.34	0.823	0–2	0–4
H–Y	2.19	0.662	0–4	0–5

## Data Availability

The speech data analyzed in this study were drawn from the publicly available PC-GITA corpus (Orozco-Arroyave et al., 2016 [39]). The Phonet toolkit used for feature extraction is also publicly available. Derived acoustic measures (posterior probabilities, harmonics-to-noise ratio values) and R Markdown analysis scripts are available at https://osf.io/cvuhw/files (accessed on 26 October 2025).
